# Glycopeptide antibiotic drug stability in aqueous solution

**DOI:** 10.1186/s41120-022-00067-0

**Published:** 2022-12-12

**Authors:** Sardar M. Jakaria, David E. Budil, James Murtagh

**Affiliations:** 1 Hikma Pharmaceuticals, Bedford, OH 44146 USA; 2 Department of Chemistry and Chemical Biology, Northeastern University, MA 02115 Boston, USA

## Abstract

Glycopeptide antimicrobials are a class of naturally occurring or semi-synthetic glycosylated products that have shown antibacterial activity against gram-positive organisms by inhibiting cell-wall synthesis. In most cases, these drugs are prepared in dry powder (lyophilized) form due to chemical and physical instability in aqueous solution; however, from an economic and practical point of view, liquid formulations are preferred. Researchers have recently found ways to formulate some glycopeptide antibiotic therapeutic drugs in aqueous solution at refrigerated or room temperature. Chemical degradation can be significantly slowed by formulating them at a defined pH with specific buffers, avoiding oxygen reactive species, and minimizing solvent exposure. Sugars, amino acids, polyols, and surfactants can reduce physical degradation by restricting glycopeptide mobility and reducing solvent interaction. This review focuses on recent studies on glycopeptide antibiotic drug stability in aqueous solution. It is organized into three sections: (i) glycopeptide antibiotic instability due to chemical and physical degradation, (ii) strategies to improve glycopeptide antibiotic stability in aqueous solution, and (iii) a survey of glycopeptide antibiotic drugs currently available in the market and their stability based on published literature and patents. Antimicrobial resistance deaths are expected to increase by 2050, making heat-stable glycopeptides in aqueous solution an important treatment option for multidrug-resistant and extensively drug-resistant pathogens. In conclusion, it should be possible to formulate heat stable glycopeptide drugs in aqueous solution by understanding the degradation mechanisms of this class of therapeutic drugs in greater detail, making them easily accessible to developing countries with a lack of cold chains.

## Introduction

**Fig. 1:**
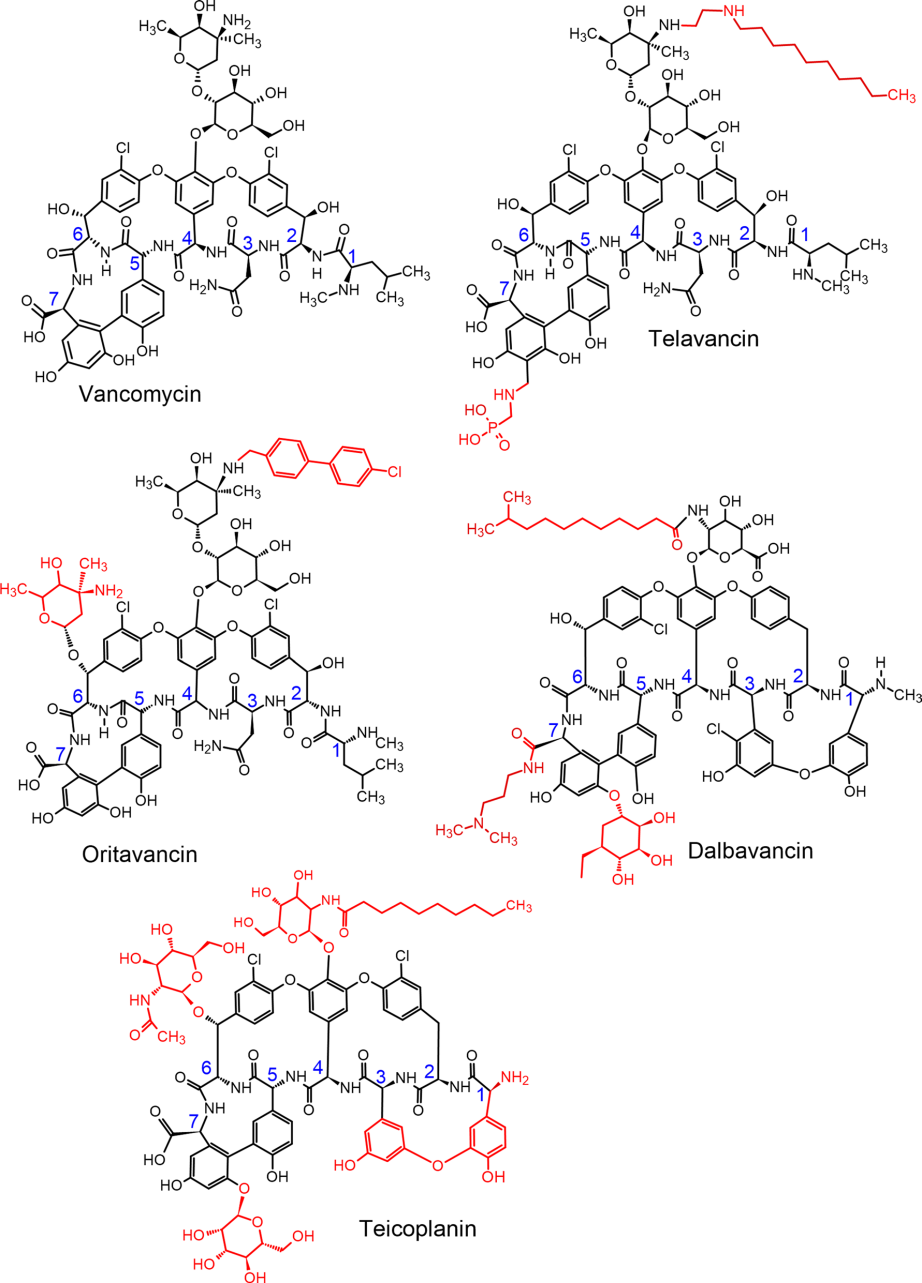
Structures of glycopeptide and lipoglycopeptide antibacterial drugs included in this review. The shared cyclopeptide frame of the drugs is shown in black, with the α-carbons of the residues numbered in blue. Key structural modifications of the basic vancomycin structure are highlighted in red [9]

The clinical discovery of glycopeptides, a class of drugs of microbial origin based on glycosylated polycyclic nonribosomal peptides [1], as a therapeutic drug class has been a revolution in the infectious disease field. The first glycopeptide, vancomycin, was discovered during what is today known as the “Golden Age of Antibiotics,” and the use of therapeutic glycopeptides has expanded rapidly since then. This class of therapeutic drugs has rapidly grown in the drug market based on its higher efficiency and lower toxicity compared with other synthetic antibiotic drugs. Glycopeptides are natural products; however, in the last 20 years, semi-synthetic derivatives with better activity and pharmacokinetics properties have been developed [2–5], several of which are shown in Fig. 1. The shared framework of the natural glycopeptides is a cyclic peptide containing seven amino acids and two linked glycosides [6], which is shown in black in Fig. 1. Early structure function and NMR studies showed that the peptide backbone of vancomycin binds to the D-Ala-D-Ala terminus of certain peptidoglycan precursors, especially lipid II, via the formation of five hydrogen bonds, as summarized in references [6, 7]. A structural determination using the strategy of crystallizing a carrier protein bound to dalbavancin via an analog of the D-Ala-D-Ala substrate [8] showed that the drug adopts a folded-leaf structure around the substrate with residues 1–3 on one side and residues 5–7 on the other. The shared features of the cyclic peptide core of all the compounds shown in Fig. 1 suggest they all bind the D-Ala-D-Ala group in a similar fashion.

Various modifications of the basic vancomycin structure, shown in red in Fig. 1, increase antibacterial potency and drug half-life in different ways. For example, the chlorine and sugar groups in oritavancin promote the formation of drug homodimers, resulting in the cooperative binding of the drug to the target [7, 10]. Interactions with the bacterial membrane have been increased semi-synthetically by modifying the lipophilic side chain of the drug [9].

Physical and chemical instability may significantly degrade glycopeptide drugs during the processing and storage of aqueous formulations [11]. Physical instability leads to processing problems such as clogged filters and precipitated solutions. Chemical instability leads to degradation during solution storage or processing. Due to their instability, these drugs must be stored at low temperatures during shelf storage and in transport. Therefore, such drugs are not easily accessible in developing countries, especially in rural and tropical areas lacking a cold chain [12, 13]. Freeze-dried (lyophilized) formulations are the preferred method to overcome peptide instability issues [14]. This is an expensive method due to high manufacturing costs and storage volumes, including vacuum-sealed vials of lyophilized powder and vials of sterile water for reconstitution.

Therefore, an aqueous preparation is preferable for peptide or glycopeptide drugs. However, stabilizing the peptide or glycopeptide drugs in aqueous formulation remains a challenge. The degradation products of peptides or glycopeptides are of concern owing to their potential for biological activity or therapeutic effects in competition with their parent drug molecule [15]. Several studies and reviews have been conducted regarding protein and peptide degradation; however, the degradation pathways of glycopeptide antibiotics currently in the market have not been characterized in depth [16, 17]. Physical instability such as adsorption [18] and aggregation, as well as chemical degradation such as hydrolysis, oxidation, and deamidation [19], are observed in peptide drugs. Information has been published on the instability and possible degradation pathways of peptides, including strategies for stabilizing them in aqueous preparations [16]. In general, peptide drugs must be stored and transported under a cold chain regimen [20].

Studies of peptide drug stabilization strategies thus provide a useful point of departure for considering the stability of glycopeptides. In this review, we discuss the factors that affect the chemical and physical integrity of glycopeptides in aqueous solution. The main difference between peptides and glycopeptides are the carbohydrate groups (glycans) linked to the side chains of the amino acid residues of the peptide core. Currently marketed glycopeptide drugs are mostly formulated in dry powder (lyophilized) form and stored at 2–8 °C, as shown in Table 1.

**Table 1:** Therapeutic glycopeptide-based products in the USA

Generic names	Trade names	Commercial source	Dosage forms	Shelf life and storage cond.	pH (adj. agent)	Product Information
Vancomycin	VANCOCIN VANCOMYCIN HCl Pulvules Firvanq	AMI Pharmaceuticals, Inc. (VANCOCIN capsules) Mylan Institutional, LLC (VANCOMYCIN HCL powder)	Capsule, powder for oral, powder for injection, and injection solution	2 y at 2–8 °C	2.5–5.5 (HCl or NaOH)	[21, 22]
Telavancin	VIBATIV	Theravance Biopharma, Inc.	Powder for injection	3 y at 2–8 °C	4.0–5.0	[23]
Dalbavacin	DALVANCE	Allergan Pharmaceutical Company (Durata Therapeutics Inc.), Vicuron Pharmaceuticals LLC	Powder for injection	3 y at up to 25 °C	4–5 (NaOH or HCl)	[24, 25]
Oritavancin	ORVACTIV	Melinta Therapeutics	Powder for injection	3 y at up to 25 °C	3.1–4.3 (H_3_PO_4_)	[26]
Teicoplanin	TARGOCID (European Market only)	Sanofi–Aventis	Powder for injection	3 y at 2–8 °C	7.2–7.8 (NaOH)	[27]

## Results

### Glycopeptide instability and degradation pathways

The integrity of glycopeptide drugs is affected by formulation, processing, storing, and shipping conditions. Like peptides, glycopeptides in aqueous solution undergo numerous degradation pathways that can be classified as chemical or physical instability. Chemical degradation of glycopeptides includes the types of bond cleavage undergone by peptides, such as hydrolysis, oxidation, and deamidization. In addition, their carbohydrate moieties may undergo racemization and isomerization reactions. Glycosylation may also modulate the precipitation and/or aggregation properties of glycopeptides relative to peptides. In this section, we review the known chemical and physical degradation pathways specific to glycopeptides.

### Hydrolytic pathways

#### Acid-/base-catalyzed hydrolysis

Glycopeptide stability in aqueous solution depends strongly on pH. It is reported that vancomycin undergoes base-catalyzed hydrolysis at pH 8 to form a succinimide degradant (succinimide 11) [28]. Between pH 1 and 3, the acid-catalyzed reaction of vancomycin has been observed in aqueous solution [29]. The water-catalyzed reaction has been observed between pH 3 and 5.7, and between pH 5.7 and 7.0, vancomycin degradation was faster in phosphate-buffered solution, where HPO_4_^2-^ (K_5_) had the dominating catalytic effect [29] and K_5_ was the second order rate constant for the acid/base catalysis of vancomycin hydrochloride.

#### Deamidation of asparagine

**Fig. 2:**
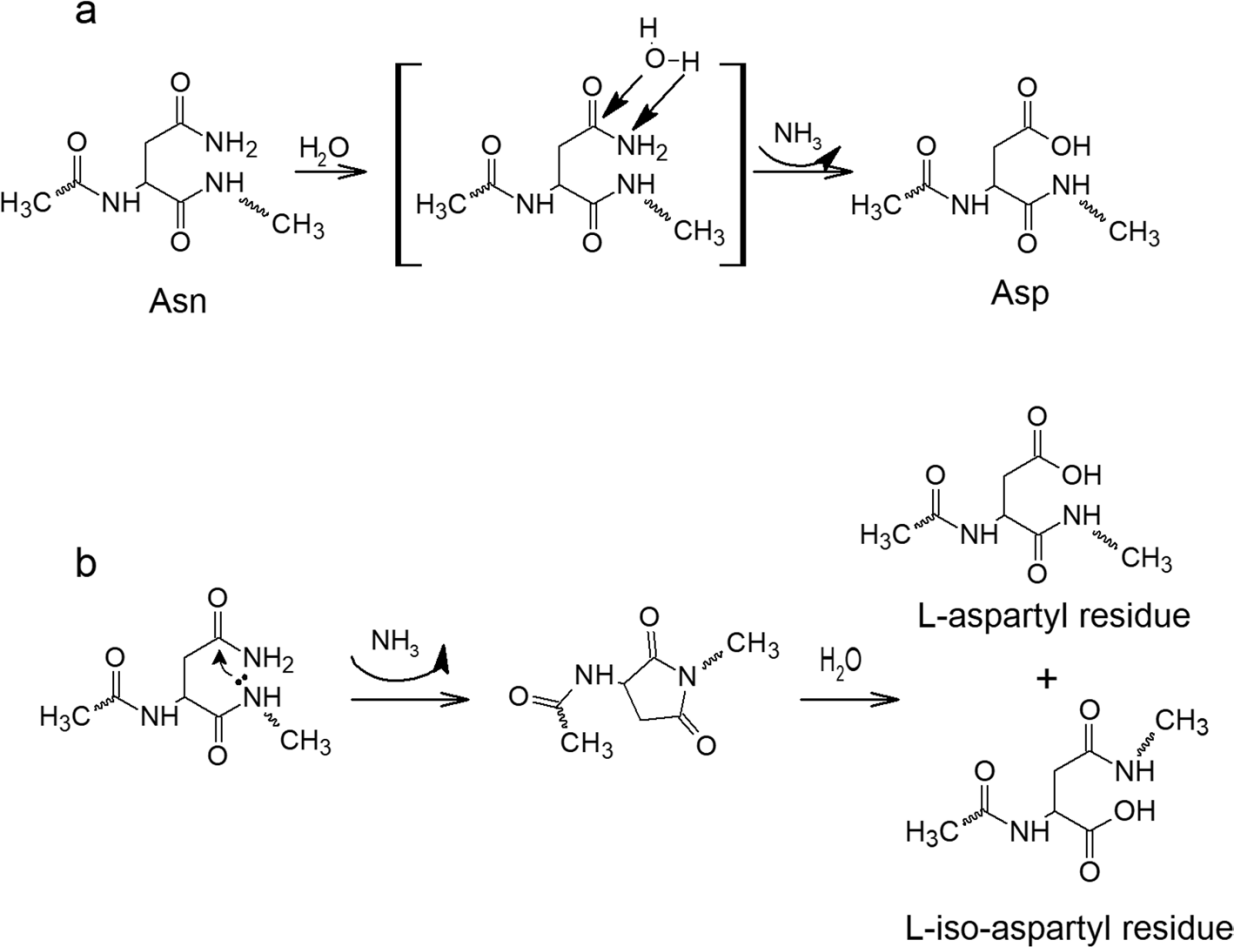
Mechanisms for the deamidation of the asparagine residue via (**a**) direct hydrolysis and (**b**) formation of a succinimide intermediate [16, 28, 32]

Peptides and glycopeptides containing the asparagine (Asn) residue readily undergo deamidation to form aspartic acid (Asp) under physiological conditions. Deamidation of asparagine can occur under acidic, basic, and neutral conditions. Under acidic conditions, the Asn side chain amide is directly hydrolyzed to form Asp; however, under basic and neutral conditions, a cyclic imide intermediate can be formed through an intermolecular reaction in which the carbonyl carbon of the Asn side chain is attacked by the backbone amide nitrogen of the following residue (Fig. 2b). The deamidation rate thus depends on the peptide’s amino acid sequence [30], with the highest rates observed when Asn is followed by glycine (Gly), alanine (Ala), serine(Ser), or Asp [31]. Direct hydrolysis or succinimide-mediated deamidation can also occur in peptide or glycopeptide drugs in aqueous solutions [32]. It is noteworthy that both reactions can occur in the absence of acid or base catalysis, as shown in Fig. 2 [16, 32].

### Oxidative degradation

#### Chemical oxidation

**Fig. 3:**
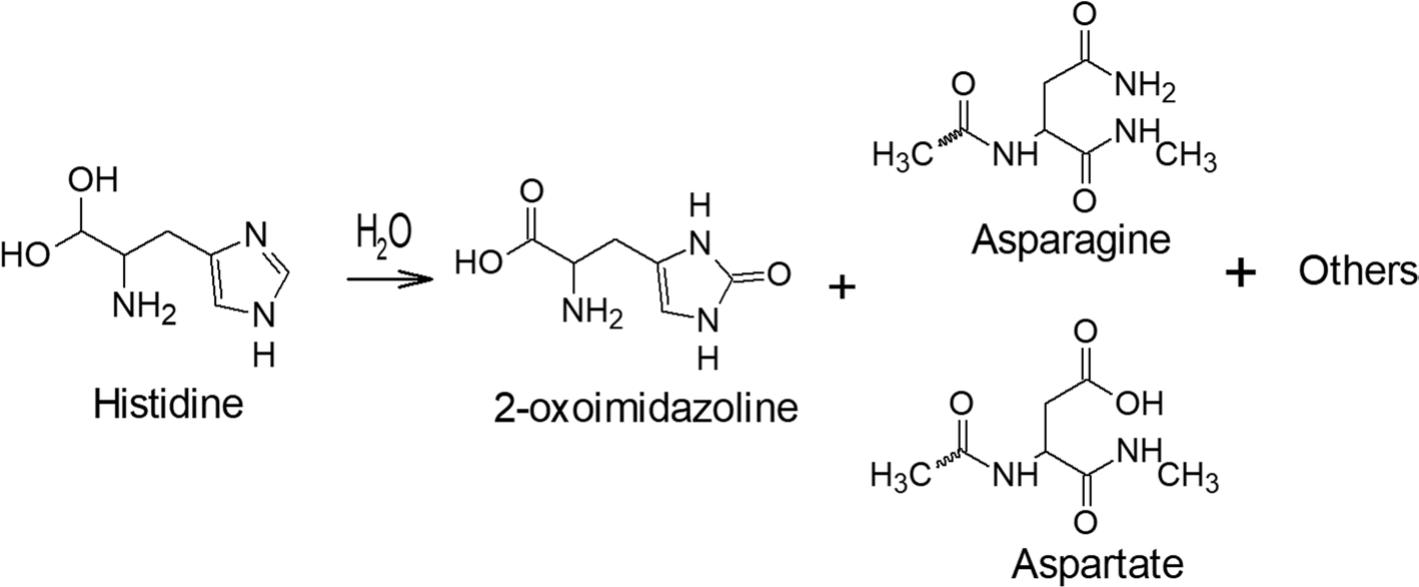
Oxidation reaction of histidine through an oxometallocyclic intermediate to form various degradation products, such as 2-oxomidazoline, asparagine, and aspartate [35]

Oxidation typically increases the electronegative atoms in a molecule, thereby producing oxygen or halogens as the electronegative heteroatoms [33]. Reactive oxygen species may react with amino acid residues such as cysteine (Cys), methionine (Met), histidine (His), tyrosine (Tyr), and tryptophan (Trp) in a peptide or glycopeptide. His, Tyr, and Trp undergo attack on their aromatic rings, whereas Cys and Met are attacked at their sulfur atoms [34]. The mechanism of His oxidation reaction is shown in Fig. 3 [16, 35].

#### Light-induced oxidation

Light-induced oxidation can occur due to the absorption of ultraviolet light by aromatic side chains of a peptide or glycopeptide. As a result, the molecule is raised to an excited state, and energy transfer from the molecule to molecular oxygen converts oxygen to its reactive singlet state. Tyr can react to produce mono, di, tri, and tetrahydroxyl Tyr by-products [36]. In addition, peptide or glycopeptide aggregation can occur due to the crosslinking of oxidized Tyr residues [37].

### Aggregation and precipitation

Aggregation of drug molecules may involve noncovalent forces, including ionic and hydrophobic interactions. There are five pathways via which proteins or peptides can form an aggregate, including (i) association of native monomers, (ii) aggregation of conformationally altered monomers, (iii) aggregation of chemically modified monomers, (iv) nucleation-controlled aggregation, and (v) surface-induced aggregation [38]. It is possible that precipitation can occur at higher peptide concentrations during shelf life due to these factors [39]. Peptide gel formation can also occur depending on the peptide’s amino acid sequence, concentration, interactions with salts in solution, temperature, time, pH, and agitation [40–42].

### Factors to improve glycopeptide stability in aqueous formulations

Based on the above discussion, it is obvious that knowledge of a glycopeptide’s structure and its degradation pathways is key to improving its stability in aqueous solutions. The known degradation pathways, stabilization strategies, and relevant amino acid residues [16] for peptide and glycopeptide molecules are shown below.

#### Improving hydrolytic stability

##### Buffer species, pH, and oxidative optimization

The pH of a solution is the most critical factor for glycopeptide stability in aqueous formulations. For slow intravenous administration and other parenteral routes, the acceptable pH ranges are 3–10.5 and 4–9, respectively, to alleviate discomfort at the injection site [43, 44]. Hence, it is critical to study the pH range of 3–10 with different buffers during the development of a glycopeptide drug formulation [45, 46]The pH should not be less than 3.0 to avoid any direct hydrolysis of Asn side chains and to minimize hydrolytic fragmentation [38]. Deamidation is sensitive to buffer composition and concentration as well as to pH. Glycopeptide and peptide formulations with pH between 3 and 5 minimize deamidation [47–51], but hydrolysis of the Asn side chain amide can also be inhibited below neutral pH.

It is noteworthy that increased ionic strength can have either a stabilizing or a destabilizing effect, depending on the nature of the charge–charge interactions within the peptide [52–54]; however, there is no effect on deamidation or hydrolysis in small peptides [55]. Oxidative reactions can be caused by the presence of oxygen, metal ions, or light. To inhibit oxidation reactions, the use of antioxidants in solution and primary and secondary packaging is recommended to protect the sample from light and oxygen. Lower pH reduces oxidation of peptides containing Tyr and Trp [35]. Addition of an antioxidant such as bisulfite to the solution frequently results in unwanted reactions as it is a strong nucleophile and can react with peptides. Chelating agents such as EDTA, DTPA, O-hydroxyphenylacetic acid, TRIS, citric acid, and tartaric acid can be used to inhibit oxidation via complexation of copper trace metal ions, and desferal, DTPA, inositol hexaphosphate, and ethylenediamine bis(o-hydroxy-phenylacetic acid) can be used for iron ions [56]. Polyols such as mannitol, trehalose, sucrose, maltose, and raffinose can also be used to prevent peptide oxidation [57, 58].

##### Cosolvent effects

Polyhydric alcohols and carbohydrates can be used to reduce peptide degradation; polyols such as propylene glycol can improve stability with respect to hydrolysis [56]. Glycerol [55] and ethanol [59] can reduce deamidation in peptide aqueous formulations. Low molecular weight cosolvents such as polyethylene glycol (PEG) can also attenuate hydrolysis by reducing the aggregation of peptides[14, 39, 60].

#### Inhibiting aggregation and precipitation

Optimization of pH and ionic strength and the use of sugars, amino acids, and/or polyols, cyclodextrins, and surfactants can inhibit dimerization, aggregation, and precipitation [38, 61–63] Examples from the literature include the use of divalent metal ion salts such as MgCl_2_, CaCl_2_, and ZnCl_2_ with citrate buffer to inhibit Cys-mediated dimerization of oxytocin [64]. Polysorbate 20 and 80 reduce agitation-induced aggregation [65, 66]. Amino acids such as asparagine, glutamic acid, glycine, arginine, and lysine have been reported to reduce aggregation[67–69]. Extremolytes [70], such as polyol derivatives ectoin and hydroxyectoin [71], betain [72], trehalose [73], proline, and mannosylglycerate [74], can also be used to stabilize peptides. Extremolytes form solute hydrate clusters that are preferentially excluded from the hydrate shell of the peptide due to repulsive interactions between the extremolyte and peptide backbone. Water accumulation near peptide domains forms a compact structure with a reduced surface area that minimizes interactions, leading to aggregation [75–77].

### Degradation pathways and formulation of currently marketed glycopeptide drugs

The available current research on each therapeutic glycopeptide drug (Table 1) will now be reviewed and discussed in terms of the degradation pathways summarized above.

#### Vancomycin

Vancomycin is the oldest and therefore one of the most commonly used glycopeptide antibiotics [1, 78, 79]. It is recommended for skin, bloodstream, and various bone and joint infections. The increasing prevalence of methicillin-resistant *Staphylococcus aureus* (MRSA) infections has made vancomycin the treatment of choice for such conditions, and it has been used extensively. However, the overuse of this antibiotic has produced *S. aureus* strains with reduced susceptibility to vancomycin, thereby compromising its antistaphylococcal activity and threatening to render vancomycin useless as a therapeutic [80].

**Fig. 4:**
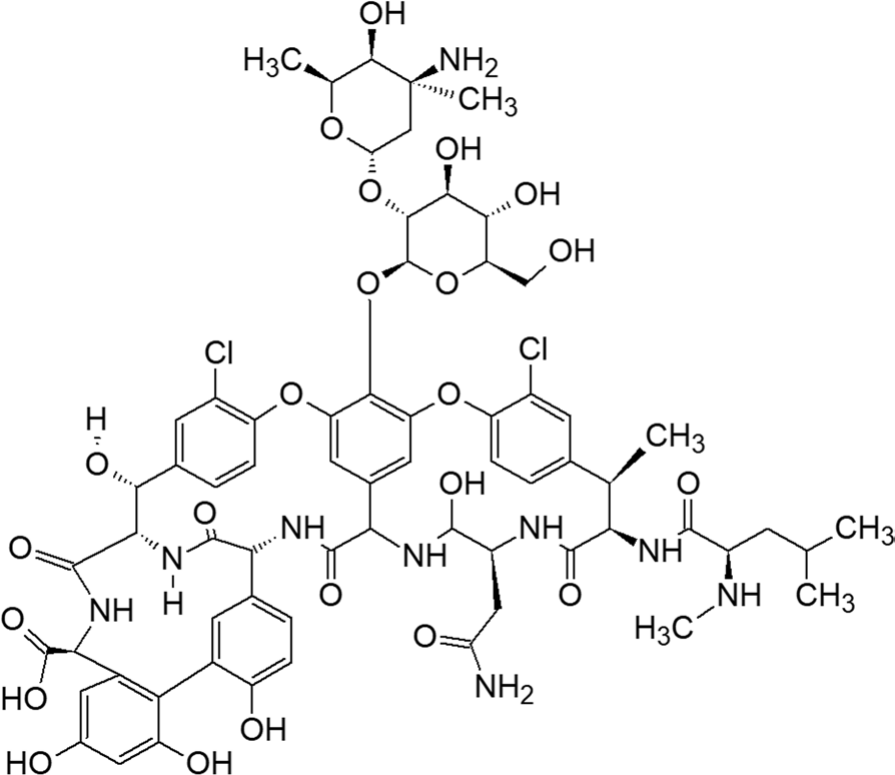
Structural formula of vancomycin [83]

A useful pharmacodynamic metric for vancomycin’s effectiveness in treating *S. aureus*, including methicillin-susceptible *S. aureus* (MSSA), MRSA, and vancomycin-intermediate *S. aureus* (VISA) strains, is the ratio of area under curve (AUC) to minimum inhibitory concentration (MIC) as measured in neutropenic mouse models [81]. An AUC/MIC ratio of ≥ 400 proves the clinical effectiveness of vancomycin [81]. Studies show that in vivo isolates of the drug develop decreased susceptibility but not of sufficient magnitude to cross a breakpoint threshold [81]. The resistance may be unstable and could result from selective processes that occur in MRSA clinical strains during vancomycin therapy, leading to a decrease in the apparent effectiveness of vancomycin in vivo [82]. Vancomycin is a tricyclic glycopeptide comprising a heptapeptide chain that forms a tricyclic structure along with an attached disaccharide that is composed of vancosamine and glucose, shown in Fig. 4 [83]. Commercial vancomycin is a hydrochloride salt and is most soluble at pH 3–5 [84]. Its solubility decreases at higher pH values, and it is unstable in alkaline solutions [84].

**Fig. 5:**
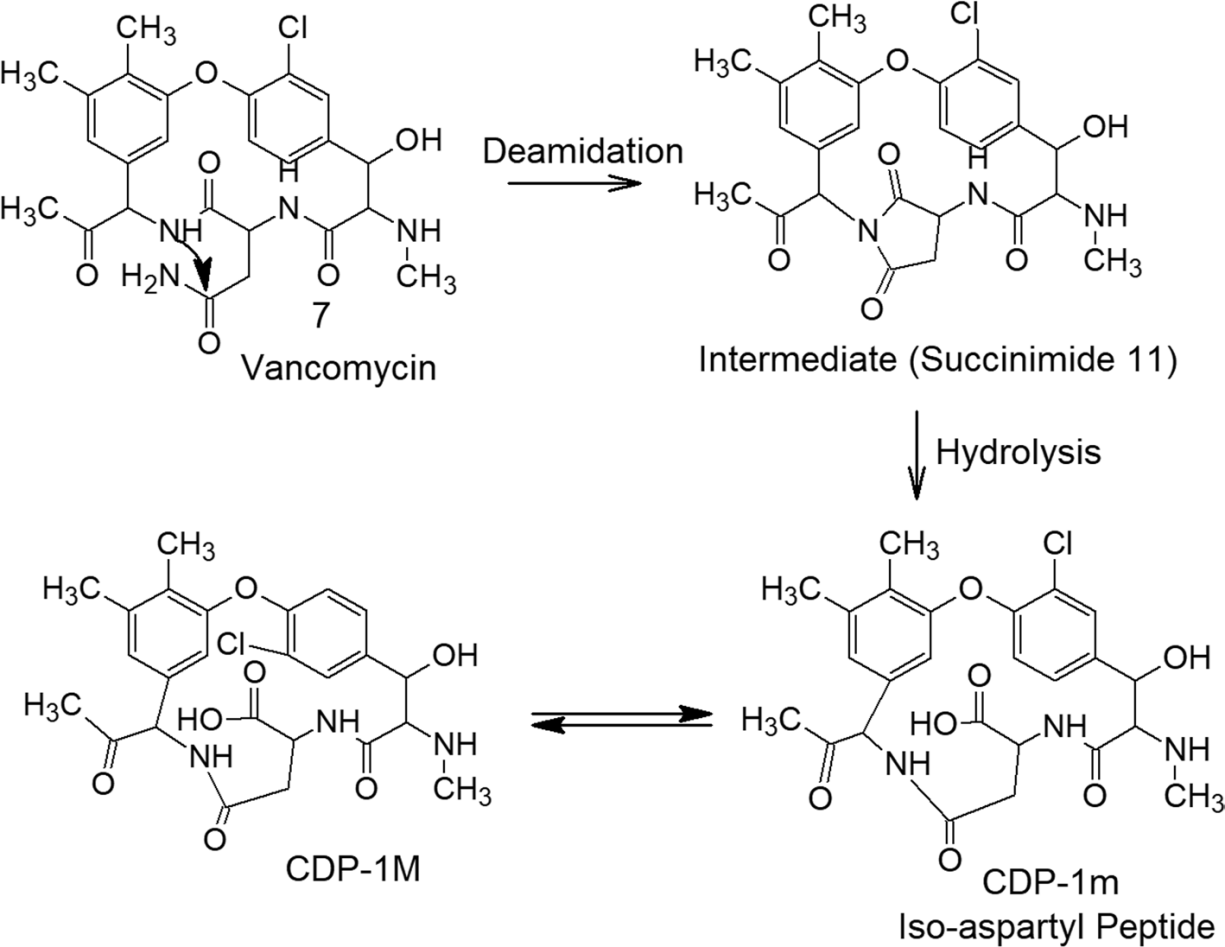
Deamidation, hydrolysis, and rearrangement of vancomycin to form degradation products [28]

Vancomycin in aqueous solution is unstable, forming degradation products that have been designated CDP-1 or CDP-1-M and CDP-1-m (collectively, the CDP-1s) [28, 84]. CDP-1 products result from hydrolysis, deamidation, and rearrangement of the asparagine moiety in the vancomycin structure, as shown in Fig. 5 [28]. These degradation products precipitate in aqueous solution and are therefore unsafe for injection. To avoid such degradation, vancomycin is formulated for therapeutic use as a dry powder in capsules for oral administration [21] and as a sterile dry powder in vials reconstituted with sterile water and diluted with dextrose or saline solution to a final concentration of 5 mg/mL [22].

A stable, ready-to-use, room-temperature aqueous solution of vancomycin comprising vancomycin, Trp, and water has been developed [84]. Trp inhibits CDP-1 degradation by forming noncovalent, reversible, and dissociable molecular complexes via hydrophobic interactions with vancomycin [84]. The optimal molar ratio of vancomycin to Trp in this complex is between 5:1 and 1:1. Another ready-to-use formulation of this glycopeptide has been stabilized below 25 ºC using a cosolvent such as PEG, N-acetyl-D-alanine, and L-lysine hydrochloride (hydrophilic amino acid) in water at a pH between 4.5 and 5.5 [84, 85]. Additionally, sulfobutylether-betacyclodextrin inhibited chemical and physical degradation of vancomycin in aqueous solution for 4 weeks at room temperature [86].

#### Telavancin

Telavancin is a second-generation lipoglycopeptide that is essentially a chemical modification of vancomycin. It has a lipophilic tail to the vancosamine sugar and contains a hydrophilic group at the 4′ position of aromatic amino acid 7. It has been approved for the treatment of gram-positive complications of bacterial pneumonia caused by *S. aureus* and is being evaluated in patients with *S. aureus* and *S. aureus* right infective endocarditis [87]. Telavancin exhibits rapid bactericidal action that is thought to arise from cooperative binding of the nascent peptidoglycan to the acyl-D-Alanyl-D-Alanine subunit in combination with the insertion of its lipophilic tail into the bacterial membrane. This disrupts the membrane function by inducing and depolarizing the membrane although it apparently does not cause cell lysis [87].

Resistance to telavancin is similar to that for vancomycin, namely the VanA modification of D-Ala-D-Ala to D-Ala-D-Lac, and cell-wall thickening that characterizes the VISA strain and other VISA phenotypes. In vancomycin-resistant enterococci (VREs), telavancin induces the expression of VanA but not does induce VanB in VREs carrying that gene. In addition, telavancin reduces the level of the peptidoglycan precursor with terminal D-Ala-D-Ala in the vanA strain through its effect on the D, D-dipeptidase, but this does not occur in the vanB strain [87]. High levels of telavancin resistance in MRSA, MSSA, or VREs were not observed in in vitro resistance selection studies. Continuous monitoring shows that telavancin remains active in vivo against a range of gram-positive pathogens [87, 88]. In addition, telavancin remains effective against MRSA according to the results of Phase 3 clinical trials in vivo [89]. Use of telavancin may be a potential treatment option for patients with MRSA bacteremia as salvage therapy [90].

**Fig. 6:**
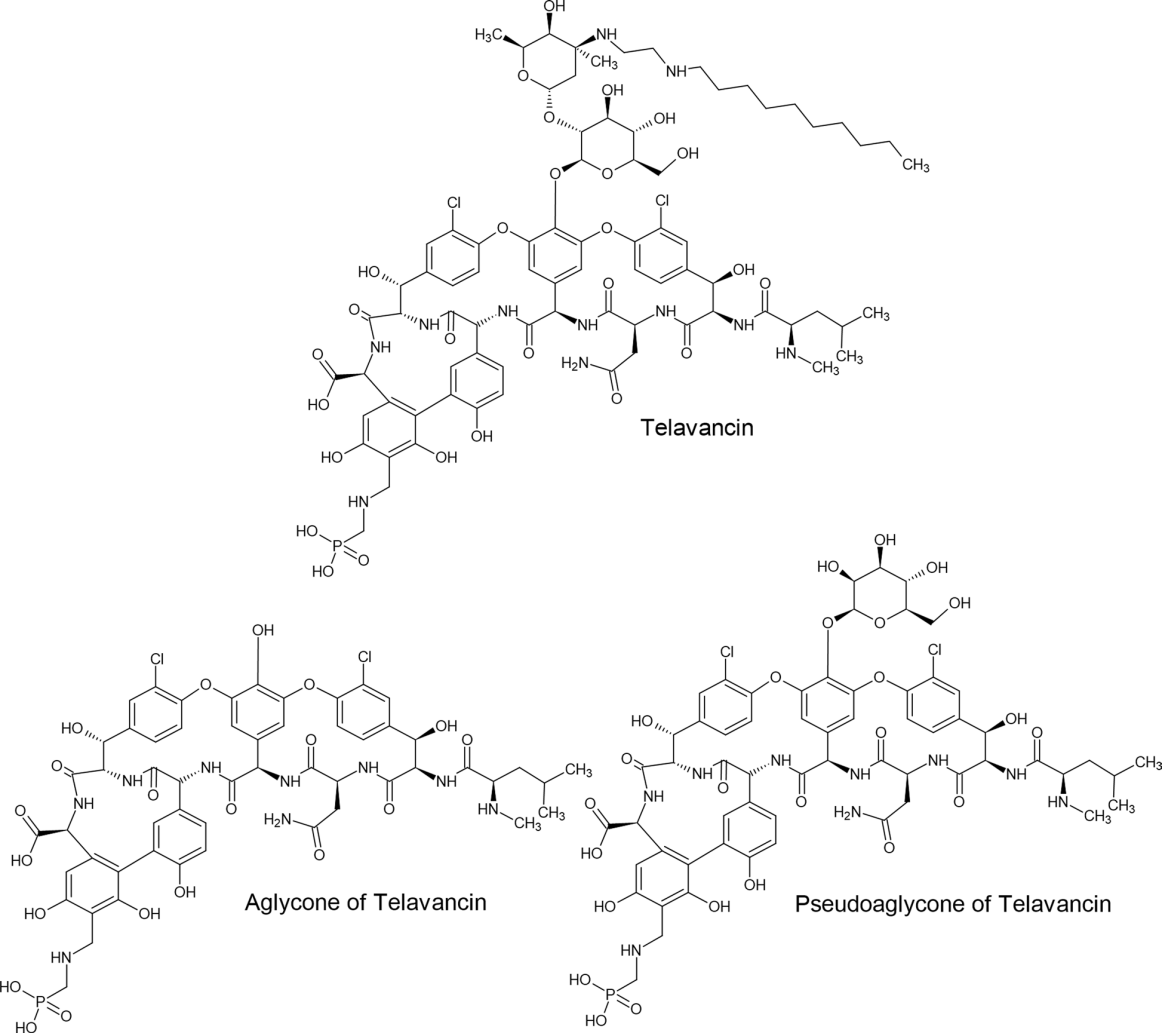
Structures of telavancin and its pseudoaglycone and aglycone degradation products [91]

At ambient temperatures, telavancin hydrochloride salt forms two principal degradation products, pseudoaglycone and aglycone, which appear as impurities of telavancin in aqueous solution, as shown in Fig. 6 [91]. The pseudoaglycone degradant develops from the hydrolysis of the lipidated vancosamine moiety, and the aglycone degradant develops from the hydrolysis of the glucose moiety of telavancin [91]. To avoid these types of degradation, telavancin is formulated for therapeutic use as a dry powder filled in vials and stabilized in hydroxypropylbetadex and mannitol. It is generally reconstituted with sterile water, 5% dextrose injection or 0.9% sodium chloride injection to 15 mg/ml, pH adjusted to between 4.0 and 5.0 with sodium hydroxide or hydrochloric acid, and subsequently diluted with 5% dextrose injection or 0.9% sodium chloride injection or lactated ringer’s injection to a final concentration of 0.6–8 mg/mL for doses less than 150 mg or greater than 800 mg. For doses of 150–800 mg, the appropriate volume of reconstituted solution as per package inserts must be further diluted in 100–250 ml prior to infusion [23].

#### Teicoplanin

**Fig. 7:**
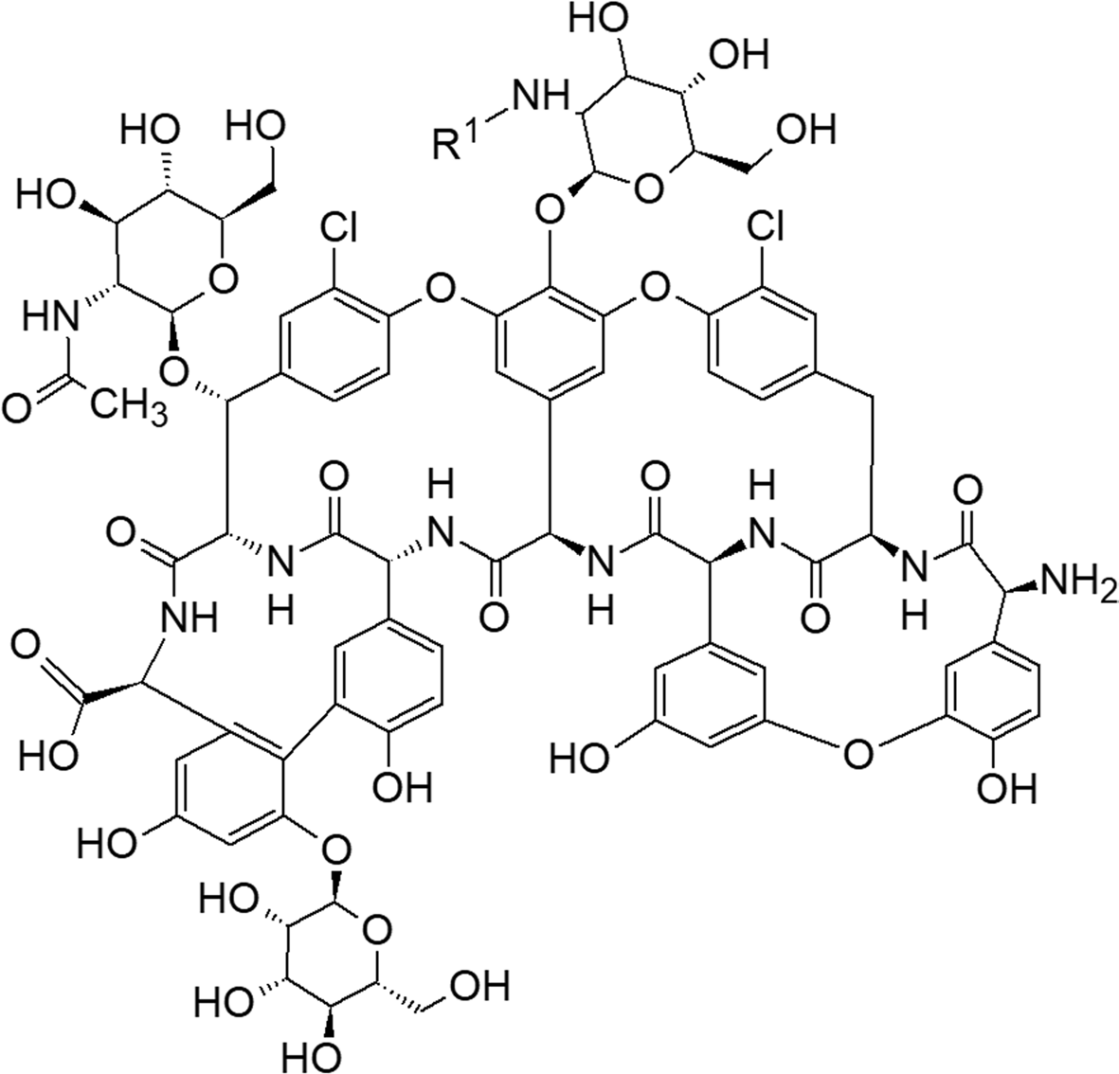
Structural formula of teicoplanin. The structures of the R group for the different major homologs of teicoplanin are given in Table 2 [92]

Teicoplanin is another semi-synthetic glycopeptide antibiotic isolated from the fermentation broth of a strain of *Actinoplanes teichomyceticus* [92], consisting of five homologs with a common aglycone [93]. The aglycone contains seven amino acids bound by peptide and ether bonds to form a four-ring system and the homologs differ in the fatty acyl side chain attached to the sugar [94, 95] (Fig. 7 and Table 2). Teicoplanin usage in treatments showed fewer patient side effects in comparison with other glycopeptide antibiotics [92]. Teicoplanin is exceptional among the vancomycin-type lipoglycopeptides in that it has a membrane-anchoring tail but does not dimerize [95].

**Table 2:** Lipophilic side chains for the different isoforms of teicoplanin according to the nomenclature of reference [92]

Teicoplanin isoform	R side chain
A_2_-1	
A_2_-2	
A_2_-3	
A_2_-4	
A_2_-5	

Teicoplanin degradation by oxidation and hydrolysis forms triphenyl ether, diphenyl ether, and diphenyl moieties [96]. Teicoplanin is formulated for therapeutic use as a dry powder filled in vials; it is generally reconstituted with sterile water to 67 mg/mL or 133 mg/mL and subsequently diluted with 0.9% sodium chloride, ringer solution, ringer-lactate solution, or injection solutions with 5% or 10% dextrose, 1.36% or 3.86% glucose, 0.18% saline with 4% glucose, or 0.45% saline with 5% glucose [27].

#### Dalbavancin

**Fig. 8:**
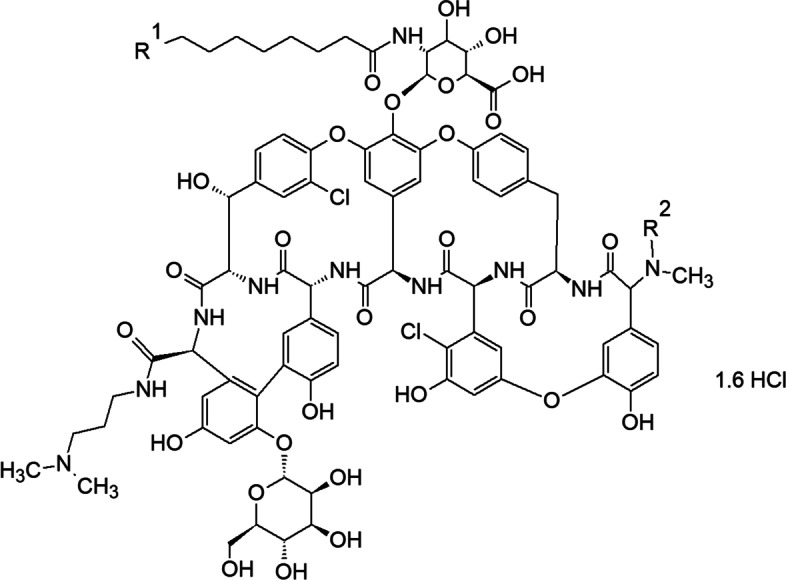
Structural formula of dalbavancin. The structures of the R_1_ and R_2_ groups for the different homologs of dalbavancin are given in Table 3 [24, 25]

Dalbavancin was developed over 15 years by four different companies before receiving final approval for clinical use in 2014. It is a second-generation teicoplanin-type glycopeptide obtained via amidation of the peptide carboxyl group of amino acid 7 of a natural glycopeptide with 3-(dimethylamino)-1-propylamine. The introduction of this substituent enhances efficacy against staphylococci, especially those that are coagulase-negative [87]. It has been approved for treating acute gram-positive bacterial skin and skin structural infections in adult patients and has been evaluated for efficacy and safety in adult patients with osteomyelitis. In addition, it is the only Food and Drug Administration–approved product for treating infections via susceptible strains of *S. aureus*, such as MRSA, *Streptococcus pyogenes*, *Streptococcus agalactiae*, and the *Streptococcus anginosus* group [97]. Like other lipoglycopeptides, dalbavancin targets the C-terminal acyl-D-Ala-D-Ala subunit of the peptidoglycan precursor. It has in vitro bacteriocidal activity against a wide range of gram-positive bacteria and an extended half-life that attributed to its interaction with serum proteins [87]. In addition, the positively charged C-terminal of dimethylaminopropyl group can interact with the negative phospholipid headgroups of the bacterial membrane. Although vancomycin-type glycopeptides dimerize cooperatively with ligand binding, the dimerization of dalbavancin is strongly anti-cooperative with ligand binding [98]. Dalbavancin is a mixture of five closely related active factors that have been designated A0, A1, B0, B1, and B2, as summarized in Table 3. Factor B0 is the main component of dalbavancin [24]. It is worth noting that these homologs share the same core structure but differ by the presence of an additional methyl group (R2) on the fatty acid side chain and/or a terminal amino group of the structure of the N-acylaminoglucuronic acid moiety (R1) [24], as depicted in Fig. 8.

**Table 3:** Substitution patterns for the homologs of dalbavancin according to the nomenclature of reference [24, 25]

Dalbavancin	R^1^	R^2^	Molecular formula	Molecular weight (Daltons)^a^
A0	CH(CH_3_)_2_	H	C_87_H_98_N_10_O_28_Cl_2_ ·1.6 HCl	1802.7
A1	CH_2_CH_2_CH_3_	H	C_87_H_98_N_10_O_28_Cl_2_ ·1.6 HCl	1802.7
B0	CH_2_CH(CH_3_)_2_	H	C_88_H_100_N_10_O_28_Cl_2_ ·1.6 HCl	1816.7
B1	CH_2_CH_2_CH_2_CH_3_	H	C_88_H_100_N_10_O_28_Cl_2_ ·1.6 HCl	1816.7
B2	CH_2_CH(CH_3_)_2_	CH_3_	C_89_H_102_N_10_O_28_Cl_2_ ·1.6 HCl	1830.7
Mannosyl aglycone [25]	N/A	H	C_70_H_69_N_9_O_22_Cl_2_^**b**^	1459.27

^a^ Specified molecular weight for anhydrous free base

^b^ Molecular Formula derived from acylglucoronamine-free dalbavancin ([25], Structure II)

Glycopeptides, particularly dalbavancin, are unstable in aqueous solution due to their glycosidic linkages [25]. The primary degradant of dalbavancin in aqueous solution is mannosyl aglycone (MAG), which is produced via hydrolysis of the glycosidic linkage. To avoid such degradation, dalbavancin is formulated for therapeutic use as a dry powder stabilized in mannitol and lactose, filled in vials, and generally reconstituted with sterile water and 5% dextrose to a concentration of 20 mg/ml followed by dilution with 5% dextrose to a final concentration of 1–5 mg/mL [24, 25]. In a 36-month study of lyophilized dalbavancin at 5 °C, MAG increased (2.9%) in proportion to the decrease in the primary dalbavancin factor B0 (3.6%). In addition, MAG increased (~ 10.2%) over 12 months at 25 °C as the primary dalbavancin factor B0 decreased (~ 8.5%); however, other dalbavancin factors such as A0, A1, B1, B2, and related substances, as well as the pH, remained unchanged, and the water content increased by 1.6% [25].

#### Oritavancin

**Fig. 9:**
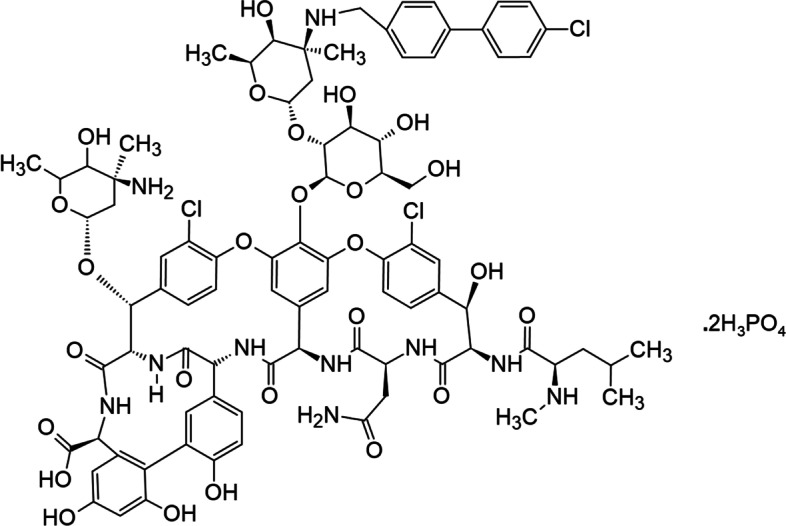
Structural formula of oritavancin [99]

Oritavancin is another second-generation semi-synthetic glycopeptide drug derived by chemically modifying the natural product chloroeremomycin and substituting N-alkyl-p-chlorophenylbenzyl on the disaccharide epi-vancosamine that is bound to the ring of amino acid 4 [87, 99]. These modifications were motivated by the vancomycin resistance reported in *Enterococcus fesium* and *Enterococcus faecalis* that motivated synthesis [99]. Oritavancin was clinically approved in 2014 for the treatment of gram-positive-related ABSSSI in adults [99]. As shown in Fig. 9, oritavancin differs from vancomycin in that it has two l-4-epi-vancosamine subunits [99] on amino acid rings 4 and 6 rather than a single l-vancosamine subunit at position 4. The addition of l-4-epi-vancosamine to chloroeremomycin provides additional benefits by enhancing antibacterial activity against vancomycin-sensitive bacteria relative to vancomycin.

Unlike telavancin and dalbavancin, oritavancin is the only lipoglycopeptide with potent activity against both VRSA and VanA-type VREs most likely because of its multiple mechanisms of action. It can also interfere with peptide transfer by binding to the pentaglycyl crosslinking segment of lipid II, thereby maintaining its affinity for the modified terminus of the peptidoglycan precursor in vancomycin-resistant strains. Furthermore, enhancement of its interaction with lipid II was achieved by fixing oritavancin to the cell membrane via the hydrophobic N-alkyl-p-chlorophenylbenzyl substituent, thereby promoting oritavancin dimerization by increasing its affinity to the target [100, 101]. Thus, the interaction of oritavancin with the membrane provides its third mechanism of action, namely, disrupting the bacterial membrane integrity by depolarization and increasing its permeability [101].

During formulation processing, oritavancin is broken down into impurities by deglycosylation, hydrolysis of amide bonds, and changes in configuration at numerous structural elements within the molecule [102]. To avoid such degradation, therapeutic oritavancin is prescribed as a dry powder that is stabilized with mannitol and filled into vials. After reconstitution with water to 10 mg/mL, the pH is usually adjusted to 3.1–4.3 with phosphate buffer and diluted with a 5% dextrose injection to a final concentration of 1.2 mg/mL [26].

## Discussion

According to the World Health Organization (WHO), drug resistance could cause 10 million deaths annually by 2050, and by 2030, antimicrobial resistance (AMR) could force up to 24 million people into extreme poverty. In 2019, the deaths of 4.95 million people were associated with drug resistant bacterial infections, and 1.27 million deaths were directly caused by AMR [103]. It is possible to reduce these deaths substantially by providing tropical countries with heat stable aqueous solution therapeutic antibiotic glycopeptide drugs. Use of lyophilized drug products is a challenge due to the absence of cold chains.

Dalbavancin (a second generation lipoglycopeptide antibiotic) is the only approved FDA product for treating infections caused by susceptible strains of *S. aureus* including MRSA, *Streptococcus pyogenes*, *Streptococcus agalactiae*, and the *Streptococcus anginosus* group [97]. In addition, dalbavancin is an emerging treatment option for most of the multidrug-resistant (MDR) and extensively drug-resistant (XDR) Gram-positive cocci bacteria [104]. Moreover, this important antibiotic lipoglycopeptide drug is also a novel candidate for COVID-19 treatment, where it blocks SARS-CoV-2 binding to its receptor ACE2 and reduces viral spread and pathogenesis in animal models [105]. Dalbavancin forms mannosyl aglycone (MAG), the hydrolyzed glycosidic linkage degradant in aqueous solution, and is therefore formulated in lyophilized form to avoid the formation of MAG. To reduce AMR deaths, especially in tropical countries due to a lack of cold chain, a heat stable injection of this important therapeutic drug is a necessity.

Besides dalbavancin, other glycopeptide therapeutic drugs such as vancomycin, telavancin, teicoplanin, and oritavancin have potential for reducing AMR deaths; however, except vancomycin, all other glycopeptides are in lyophilized form. In recent development, a stable, ready to use, room-temperature aqueous solution of vancomycin comprising, Trp, and water has been developed, where Trp inhibits CDP-1 degradation products by forming noncovalent, reversible, and dissociable molecular complexes via hydrophobic interaction with vancomycin. Another ready to use formulation has been stabilized below 25 °C using a cosolvent such as PEG, N-acetyl-D-alanine, and L-lysine hydrochloride in water at a pH between 4.5 and 5.5.

It is obvious from above discussion that a heat stable liquid solution of antibiotic glycopeptide drugs is urgently needed to reduce AMR deaths in developing countries, including dalbavancin, which is the only FDA-approved drug for MRSA, *Streptococcus pyogenes*, *Streptococcus agalactiae*, and the *Streptococcus anginosus* group, as well as a promising treatment option for most Gram-positive cocci with MRD/XRD.

## Conclusion

Most glycopeptides in aqueous solutions are unstable for long-term storage. However, a careful, thorough understanding of each glycopeptide structure and its degradation pathways such as hydrolysis of the glycosidic bond in most cases, oxidation, and deamidation or deamination should help to design formulations with specific buffer, sugar, amino acids, and excipients to significantly overcome the challenges of chemical and physical degradations. The growing number of AMR deaths predicted by 2050 makes it essential to develop a heat-stable glycopeptide antibiotic therapeutic drugs in aqueous solution that can reduce death rates worldwide, particularly in tropical countries. Aqueous solutions of vancomycin, the oldest antibiotic glycopeptide, are stable at room temperature, although other glycopeptides are yet to be evaluated. In order to formulate this important class of pharmaceuticals for heat stability in aqueous solutions and easy accessibility in developing countries without cold chains, a deeper understanding of degradation reaction mechanisms across the range of glycopeptide drugs must be developed.

The description and discussion of degradation pathways and strategies to improve the stability of aqueous solutions of glycopeptides in this review has largely been based on available literature for peptide and protein formulations. Because glycopeptides differ from proteins in several important respects, a complete understanding of glycopeptide drug degradation mechanisms of glycopeptide drugs has yet to be reached, particularly for chemically modified natural products, which may undergo reactions quite different from proteins. Since all glycopeptide antibiotic drugs contain the basic core structure of vancomycin, the development of a heat stable liquid solution for any one of them should provide better strategies for improving aqueous formulations for the entire family of glycopeptide antibiotic drugs.

## Data Availability

Not applicable.
